# Megaloappendix: A Case Report

**DOI:** 10.1155/2011/729304

**Published:** 2011-08-07

**Authors:** Abdel Hady A. Samaha, Ayman S. Tawfik, Tariq O. Abbas, Ashraf Abdelhamid

**Affiliations:** ^1^Pediatric Surgery Department, Hamad General Hospital, P.O. Box 3050, Doha, Qatar; ^2^Pediatric Surgery Department, Al-Azhar Univeristy, Egypt

## Abstract

The vermiform appendix is an organ that can have variable sizes, locations as well as functional potentials. We describe here the longest and largest appendix removed measuring about 55 cm in length.

## 1. Introduction

The vermiform appendix is an organ that can vary in size, site, and presence, as well as in other clinical and functional aspects. We describe here the longest and largest appendix removed to date, measuring about 55 cm in length.

## 2. Case Report

A 13-year-old male was admitted to our hospital with a history of abdominal distension that was neither painful nor associated with vomiting. On examination, he appeared pale and apathetic, but he was hemodynamically stable. A non-tender tennis-ball-sized mass was palpated in the right iliac fossa of his abdomen. Hernia orifices were free, and there was no discoloration of the abdomen. Ultrasonography of the abdomen was not conclusive. Computerized tomography of his abdomen showed thickened bowel walls with features also suggestive of lymphoma. The patient was kept under observation and managed conservatively. The abdominal mass decreased in size over the next week, and the patient was then discharged home. 

Four days later; however, he developed severe abdominal pain while playing football and was again transferred to the hospital. He arrested during transfer but was resuscitated and later revived. Upon arrival, he was unconscious, severely hypotensive and tachycardic, with oxygen saturation of 50%. His abdomen was severely distended, his WBC count was 15000 c/mL, and computerized tomography showed free peritoneal fluid and features suggestive of pneumonia.

**Figure 1:**
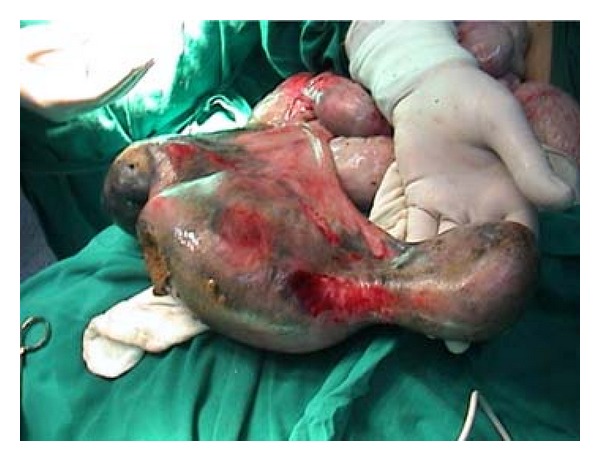
Intraoperative view showing the appendix that measured about 55 cm and was full of fecal materials.

**Figure 2:**
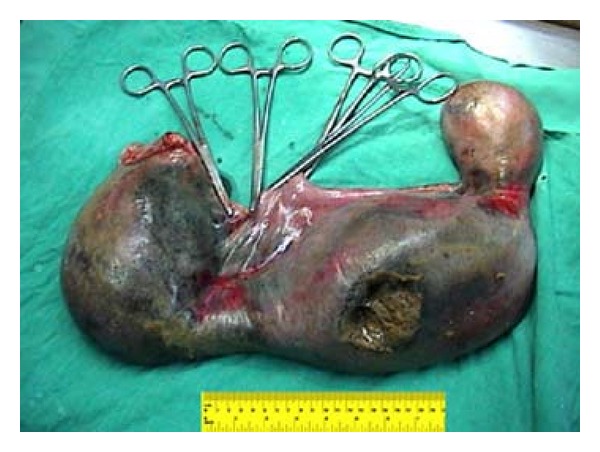
The appendix was perforated in the middle portion.

Operative exploration showed a huge and perforated appendix (Figure 1). The appendix measured about 55 cm in length, was filled with fecal material, and had a perforation in its center (Figure 2). Histopathological examination of the removed specimen confirmed an inflamed appendicular tissue.

## 3. Discussion

The vermiform appendix can vary in size and site and may be present or absent in individuals. In humans, the appendix is longer in children than in adults, becoming even smaller after midlife [1]. Moreover, about 1 in 100,000 humans are born without an appendix, and rare individuals have been reported to be born with two appendixes [1].

In humans, the appendix averages 6 to 9 cm in length. It is typically longer in males than in females. The diameter of the appendix is usually only between 7 and 8 mm and may be partially or completely closed after midlife. The longest appendix reported to date measured 26 cm (10.24 in) when it was removed at autopsy from a 72-year-old man (Guinness 2007). 

Throughout medical history, many possible functions for the appendix have been suggested, including exocrine, endocrine, and neuromuscular functions [2]. Darwin [3] suggested that the appendix had been used by primate ancestors of humans to digest leaves. Over time, as humans ate fewer leaves, the appendix evolved to a smaller size to make room for the stomach [3]. 

Currently it is unclear whether the lymphoid tissue in the human appendix performs any specialized function, different from that performed by the much larger amount of lymphatic tissue distributed throughout the gut. Most importantly with regard to vestigiality, there is no evidence from any mammal suggesting that the hominoid vermiform appendix performs functions beyond those of the lymphoid-rich caeca of other primates and mammals that lack distinct appendixes [4]. 

Recent findings have suggested that the appendix may harbor and protect bacteria that are beneficial in the function of the human colon [5]. The appendix may also be a haven for useful bacteria when illness flushes those bacteria from the rest of the intestines [6]. 

In England, 96% of hospital admissions for diseases of the appendix in 2002-2003 were emergency admissions [7]. The most common diseases of the appendix in humans are appendicitis and carcinoid tumors (appendiceal carcinoid) [8]. 

In comparison, the appendix removed from our patient was more than twice as long, measuring 55 cm in length, making it the largest and longest appendix removed to date.
